# Preoperative prognostic nutritional index is useful factor for predicting postoperative delirium after primary total joint arthroplasty

**DOI:** 10.1186/s12891-021-04626-6

**Published:** 2021-09-12

**Authors:** Jie Chen, Chaojun Zheng, Jinxiu Zhong, Guanglei Zhao, Jingsheng Shi, Gangyong Huang, Yibin Wei, Siqun Wang, Jie Yu, Jun Xia

**Affiliations:** 1 Department of Orthopedics, Huashan Hospital, Fudan University, 12 Mid- Wulumuqi Road, 200040 Shanghai, China; 2 Department of Orthopedics, Xingguo people’s Hospital, Ganzhou, 342400 Jiangxi China; 3 Department of Infectious Disease, Huashan Hospital, Fudan University, 200040 Shanghai, China

**Keywords:** Postoperative delirium, Malnutrition, Prognostic nutritional index, Total joint arthroplasty, Risk factor

## Abstract

**Background:**

Postoperative delirium (PD), as an acute brain failure, is widely reported as a very common postoperative complication, and it is closely associated with increased morbidity and mortality. Recently, malnutrition is reported as one of the risk factors for PD. The prognostic nutritional index (PNI) is a simple method for nutritional evaluation. However, few studies have discussed the effectiveness of PNI as a nutritional assessment in predicting PD after primary total joint arthroplasty (TJA). The aim of this study is to investigate potential risk factors including PNI for PD following primary TJA.

**Methods:**

A retrospective analysis of 994 patients was performed to identify risk factors associated with PD after primary TJA by using univariate and multivariate analyses. A receiver operating characteristic curve and the area under the curve were applied to evaluate the significant results of the multivariate analysis and the optimal cutoff value (CV).

**Results:**

Postoperatively, sixty-seven patients (67/994, 6.7 %) experienced PD. Univariate analysis demonstrated that operative time, duration of anesthesia, age, hypertension, serum albumin, and PNI differed between the PD and non-PD groups (*P* < 0.05). Multivariate logistic regression analysis showed that the preoperative PNI (odds ratio [OR]: 0.908; 95 % confidence interval [CI]: 0.840–0.983; CV: 47.05), age of patients (OR: 1.055; 95 % CI: 1.024–1.087; CV: 73.5 years), and hypertension (OR: 1.798; 95 % CI: 1.047–3.086), were independently associated with PD (*P* < 0.05).

**Conclusions:**

A low preoperative PNI associated with malnutrition was demonstrated to be an independent risk factor for PD following primary TJA. Patients with preoperative low PNI should be cautioned and provided with adequate nutritional intervention to reduce postoperative PD.

**Supplementary Information:**

The online version contains supplementary material available at 10.1186/s12891-021-04626-6.

## Background

Total joint arthroplasty (TJA), including total knee arthroplasty (TKA) and total hip arthroplasty (THA), is a routine and effective treatment for advanced osteoarthritis [1, 2]. While primary TJA largely has excellent clinical outcomes, severe complications can occur [3]. Among these, postoperative delirium (PD) is a serious complication after TJA, which is characterized by an acute disruption of consciousness and impairment of attention with fluctuating course [4]. The rates of PD following TJA reported in current literature range from 3.1 to 30.2 % [4–6]. As the acute state of cognitive disturbance, the onset of PD not only results in the significant delay in rehabilitation and prolonged hospitalization, but also contributes to considerable morbidity and economic burden [7, 8]. Therefore, in order to achieve optimal PD prevention after TJA, knowledge of its risk factors hold a high priority.

The impact of malnutrition on surgical outcomes following orthopedic surgery has been widely reported [9–14], and recently published studies further demonstrated that preoperative malnutrition is closely associated with PD after orthopedic surgery [12–14]. Importantly, unlike the spinal deformity that has relatively constant incidence rate [12], with the aging of society, the number of patients requiring TJA will continue to increase. Furthermore, different from the hip fracture emergency surgery [14], TJA is a selective operation, and there is enough time to adjust the preoperative nutritional status in order to reduce PD after TJA. Therefore, preoperative screening and improvements are important for patients suspected of malnutrition to avoid PD following TJA.

Recently, prognostic nutritional index (PNI), a new prognostic indicator that can be simply calculated by serum albumin and lymphocyte, have been used to evaluate both the inflammatory and nutritional status of patients accepting orthopedic or cancer surgery [12, 15, 16]. Tei et al. reported that lower PNI is a significant risk factor for PD in elderly patients with colorectal cancer [17], and Oe et al. demonstrated that a significant risk factor for delirium in 319 patients with adult spinal deformity surgeries was preoperative PNI [12]. Unfortunately, few studies have discussed the effectiveness of PNI as a nutritional assessment in predicting PD after primary TJA; however, this type of study may guide clinicians in choosing the optimal methods to prevent PD.

Therefore, the purpose of this study was to assess the incidence of PD after primary TJA and to clarify the risk factors for PD, including malnutrition identified by PNI. Furthermore, the appropriate predictive cutoffs of these factors were also analyzed in this study.

## Methods

### Subjects

**Fig. 1:**
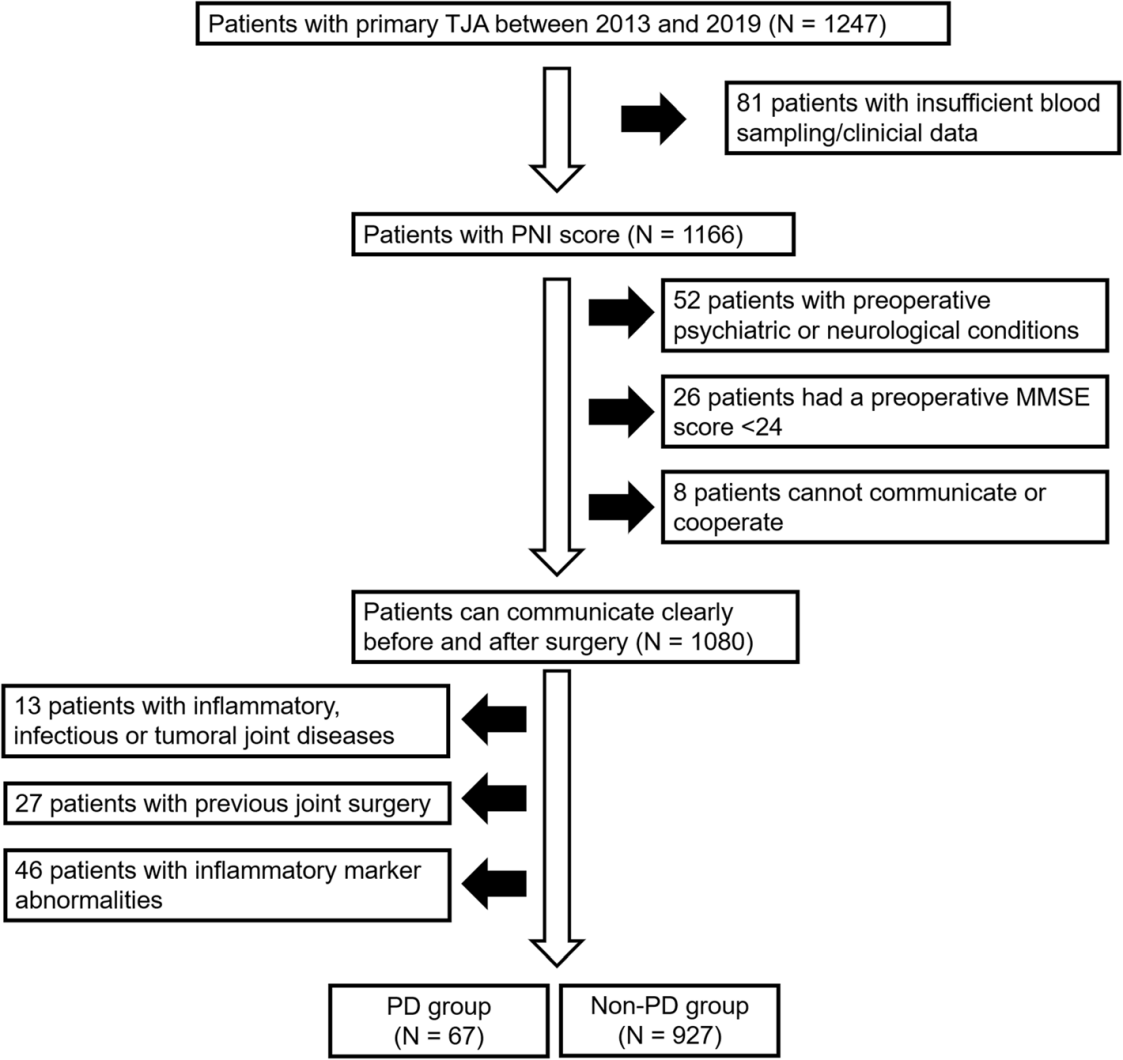
Design of the present study. TJA: Total joint arthroplasty; PNI: Prognostic nutrition index; PD: Postoperative delirium; MMSE: Mini-mental state examination; N: Number

Between January 2013 and October 2019, a total of 1247 patients accepted primary THA or TKA, and 253 of these 1247 patients were excluded since they met the exclusion criteria (Fig. 1). Therefore, 994 patients were retrospectively enrolled in this study. This study protocol was approved by the Ethics Committee of Huashan Hospital (Fudan University, Shanghai, China), and informed consent was obtained from all participants.

The inclusion criteria of this study were as follows [13, 14]: (1) patients who can communicate clearly before and after surgery, (2) follow-up period of at least 1 year to identify the postoperative complication, (3) the surgical procedure was performed by the same anesthetic and surgical team, and (4) consent to participate this study. The exclusion criteria were: (1) insufficient assessment of blood sampling and/or clinical data preoperatively; (2) surgery for the treatment of inflammatory, infectious or tumoral joint diseases; (3) with preexisting psychiatric (e.g., psychiatric, depression, etc.) or neurological conditions (e.g., dementia, delirium, Parkinson’s disease, stroke, etc.); (4) a history of medication that affect neurocognitive; (5) with other coexisting diseases that affect inflammatory marker, lymphocyte and albumin; (6) with a preoperative Mini-mental state examination (MMSE) scores less than 24; and (7) inability to cooperate or communicate.

### Perioperative management

In the present study, the patients received primary THA via a lateral approach or TKA via a medial parapatellar approach by two senior surgeons. All patients in this study accepted operation under general anesthesia combined with inhalation agents and received the same anesthesia scheme (induced anesthesia: vecuronium or rocuronium, propofol, and sufentanil; maintain anesthesia: sevoflurane inhalation, propofol, and remifentanil). Furthermore, peri-articular cocktail analgesic injection was performed, and it contained 150 mg ropivacaine and 0.5 ml adrenaline that were mixed with sterile normal saline solution to make up a combined volume of 40–50 ml. Postoperatively, oral analgesic including celecoxib 200 mg q12h was given routinely. Only for moderate or severe pain, intravenous analgesics including flurbiprofen 100 mg qd was applied. In addition, to prevent deep vein thrombosis and pulmonary embolism, the patients were asked to wear elastic stockings and perform early lower limb function exercises as part of their antithrombotic prophylactic treatments.

### Postoperative delirium assessment

In our institution, a neurocognitive evaluation was routinely performed daily (in the evening) within the postoperative 7 days (in the evening) by a same experienced clinician. The PD was defined according to Diagnostic and Statistical Manual of Mental Disorders, Fifth Edition (DSM-V, 2013) [13, 14]. Furthermore, Since 2016, both the delirium duration and severity (severity item scores of the Delirium Rating Scale-Revised-98 [DRS-R-98]) were recorded in patients with identified PD.

### Data acquisition

The measurements of patient characteristics were collected from both medical records [e.g., age, sex, height, body weight, body mass index (BMI), and American Society of Anesthesiologists (ASA) grade] and a standardized questionnaire developed for this study (e.g., alcohol consumption, current smoking, preoperative complications, and current medications; Supplementary Table 1). Surgical and anesthetic information was obtained by reviewing the operative and anesthetic records, including type of surgery, duration of operation, intraoperative blood loss, blood transfusion, and postoperative complications.

Blood sampling data were evaluated within 1 week preoperatively, including white blood cell count, lymphocyte count, neutrophils count, hemoglobin and serum albumin, and PNI was calculated by the following formula: 10 × serum albumin (g/dL) + 0.005 × total lymphocyte count (/µL) [12, 15–17]. All of these blood sampling markers were measured by the department of Laboratory Medicine, and no blood specimen was obtained solely for the purpose of this study.

### Statistical methods

SPSS 20.0 (IBM, Armonk, NY) was used to analyze the data. The Kolmogorov-Smirnov test was used to test the normality of the distributions. The measurements for non-PD and PD groups were compared using independent t-tests or Mann-Whitney tests, and the frequencies of the different measurements between these two groups were compared by Fisher’s exact test or chi-square test. Statistically significant covariates based on these univariate analyses were subjected to multivariate logistic regression analysis, and the cutoff values and area under curve (AUC) of the results of the multivariate logistic regression analysis were identified by the receiver operating characteristic (ROC) curve. Multivariate analysis of variance was used to evaluate the interaction between different independent risk factors in relation to incidence of PD, and the sensitivity, specificity, positive predictive value (PPV) and negative predictive value (NPV) of these independent risk factors were also analyzed. In the patients with PD, both duration and severity of PD were compared using Kruskal-Wallis H test among different subgroups of hypertension. Furthermore, the relationship between independent risk factors and PD was analyzed by regression analysis. A P-value of less than 0.05 was considered significant.

## Results

### Comparison between the PD and non-PD group

Among 994 patients, sixty-seven patients (67/994, 6.7 %) experienced PD, and both the delirium duration (2.7 ± 1.8 days) and severity (25.1 ± 5.9) were recorded in 39 of these 67 patients. Compared with the non-PD group, the PD group showed obviously older age, longer operative time and longer duration of anesthesia. Furthermore, a greater number of patients in the PD group had hypertension (*P* < 0.05, Table 1). Among the laboratory data, reduced serum albumin and decreased PNI were observed in the PD group compared to the non-PD group (*P* < 0.05, Table 2). In addition, a greater number of patients in the PD group presented with postoperative surgical site infection (SSI) in this study (*P* < 0.05, Table 1). Equally important, the patients with PD exhibited obviously longer hospital stay (10.7 ± 5.0 vs. 9.6 ± 3.7 days, *P* < 0.05).

**Table. 1:** Patient characteristics and surgical data between the PD and non-PD groups

	PD group	Non-PD group	P
**Number of subjects**	67	927	
**Age range (years)**	71.1 ± 9.6	66.4 ± 9.7	< 0.01*
**BMI**	24.2 ± 3.6	23.8 ± 3.6	0.41
**Sex (male vs. female)**	18 vs. 49	268 vs. 659	0.72
**Alcohol consumption**	15/67 (22.4 %)	142/927 (15.3 %)	0.13
**Current smoking**	8/67 (11.9 %)	111/927 (12.0 %)	0.99
**ASA physical status**			
I-II	40/67 (59.7 %)	611/927 (65.9 %)	0.30
III-IV	27/67 (40.3 %)	316/927 (34.1 %)	0.30
**Preoperative complications**			
Hypertension	24/67 (35.8 %)	225/927 (24.3 %)	0.04*
* Stage 1 (SBP: 140–159 mmHg; DBP: 90–99 mmHg)*	3/67 (4.5 %)	111/927	0.06
* Stage 2 (SBP: 160–179 mmHg; DBP: 100–109 mmHg)*	11/67 (16.4 %)	71/927	0.01
* Stage 3 (SBP ≥ 180 mmHg; DBP ≥ 110 mmHg)*	10/67 (14.9 %)	43/927	< 0.01
Diabetes mellitus	10/67 (14.9 %)	110/927 (11.9 %)	0.46
Heart disease	5/67 (7.5 %)	43/927 (4.6 %)	0.30
Chronic renal dysfunction	3/67 (4.5 %)	45/927 (4.9 %)	0.89
**Current medication**			
Steroid	3/67 (4.5 %)	47/927 (5.1 %)	0.83
Immunosuppressant	2/67 (3.0 %)	18/927 (1.9 %)	0.56
Anticoagulation	12/67 (17.9 %)	136/927 (14.7 %)	0.47
β-blockers	19/67 (28.4 %)	187/927 (20.2 %)	0.11
ACEIs	7/67 (10.4 %)	58/927 (6.3 %)	0.18
**Surgical data**			
Type of operation (THA vs. TKA**)**	28 vs. 39	500 vs. 427	0.05
Operative time (min)	112.1 ± 42.7	98.5 ± 38.4	< 0.01*
Anesthesia time (min)	172.8 ± 45.7	159.1 ± 42.3	0.01*
Intraoperative blood loss (ml)	308.4 ± 78.7	292.1 ± 83.6	0.11
Blood transfusion	7/67 (10.4 %)	60/927 (6.5 %)	0.21
**Postoperative complications**			
Surgical site infection	13/67 (19.4 %)	36/927 (3.9 %)	< 0.01*
Hematoma	2/67 (3.0 %)	25/927 (2.7 %)	0.89
Deep vein thrombosis	5/67 (7.5 %)	41/927 (4.4 %)	0.25
Dislocation	1/67 (1.5 %)	10/927 (1.1 %)	0.76
Periprosthetic fracture	1/67 (1.5 %)	3/927 (0.3 %)	0.14

*Statistically significant difference between the PD and non-PD groups

*PD* Postoperative delirium; *BMI* Body mass index; *PNI* Prognostic nutritional index; *TKA* Total knee arthroplasty; *THA* Total hip arthroplasty; *ASA* American society of anesthesiologists; *ACEIs* Angiotensin-converting enzyme inhibitors; *P* *P*-value

**Table. 2:** Preoperative laboratory measurements between the PD and non-PD groups

	PD group	Non-PD group	P
**Number of subjects**	67	927	
White blood cell (x10^9/L)	6.6 ± 1.7	6.7 ± 1.7	0.70
Hemoglobin (g/L)	128.8 ± 12.4	129.1 ± 13.7	0.86
Neutrophils count (x10^9/L)	4.1 ± 1.3	4.0 ± 1.4	0.64
Serum albumin (g/L)	37.7 ± 3.6	39.9 ± 3.4	< 0.01*
Lymphocyte count (x10^9/L)	1.8 ± 0.8	2.0 ± 0.7	0.06
PNI	46.7 ± 4.1	49.9 ± 5.0	< 0.01*

*Statistically significant difference between the PD and non-PD groups

*PD* Postoperative delirium; *PNI* Prognostic nutritional index; *P* *P*-value

### Risk factor analysis of PD

All statistically significant measurements between the PD and non-PD groups based on the univariate analysis, including age, operative time, duration of anesthesia, cases with hypertension, serum albumin and PNI were subjected to multivariate logistic regression analysis. The results of multivariate analysis exhibited that PNI (odds ratio [OR]: 0.908; 95 % confidence interval [CI]: 0.840–0.983; B: -0.096; *P* = 0.017), age (OR: 1.055; 95 % CI: 1.024–1.087; B: 0.053; *P* = 0.001), and cases with hypertension (OR: 1.798; 95 % CI: 1.047–3.086; B: 0.586; *P* = 0.034) were independently associated with PD. Furthermore, there is an interaction between hypertension and PNI/age in relation to incidence of PD (*P* < 0.05), and there in no interaction between PNI and age in relation to incidence of PD (*P* > 0.05).

**Fig. 2:**
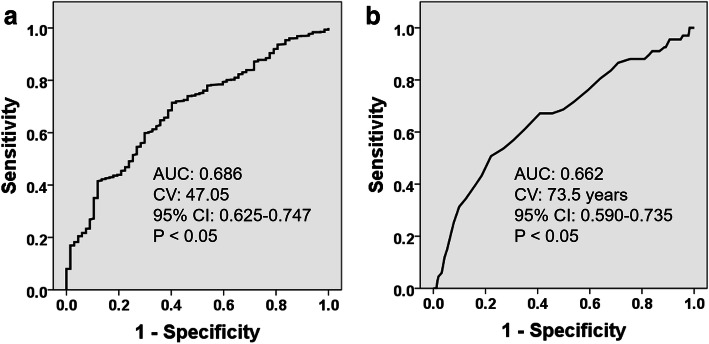
Receiver operating characteristic curve for the risk factor of PNI (**A**) and age (**B**). PNI: Prognostic nutrition index; AUC: Area under curve; CV: Cutoff values; CI: Confidence interval; P: *P*-values

The sensitivity, specificity, PPV and NPV of cases with hypertension in predicting PD after primary TJA were 35.8 %, 75.7 %, 9.6 and 94.2 %, respectively. Furthermore, ROC curve analysis revealed the cutoff values of PNI (cutoff value: 47.05; AUC: 0.686; 95 %CI: 0.625–0.747; sensitivity: 59.7 %; specificity: 71.4 %; PPV: 13.1 %; NPV: 96.1 %) and age (cutoff value: 73.5 years; AUC: 0.662; 95 %CI: 0.590–0.735; sensitivity: 50.7 %; specificity: 77.9 %; PPV: 13.1 %; NPV: 95.6 %) for predicting PD after primary TJA (*P* < 0.001; Fig. 2).

Subgroup analysis of different grades (1–3) of hypertension demonstrated that patients with moderate to severe hypertension are more likely to show PD compared with patients with mild hypertension (Table 1). Importantly, patients with stage 2 (Systolic blood pressure [SBP]: 160–179 mmHg; Diastolic blood pressure [DBP]: 100–109 mmHg) and stage 3 (SBP ≥ 180 mmHg; DBP ≥ 110 mmHg) hypertension presented with relatively longer delirium duration and higher DRS-R-98 scores than the patients with stage 1 (SBP: 140–159 mmHg; DBP: 90–99 mmHg) hypertension or cases without hypertension (*P* < 0.05). There is no difference of both delirium duration and severity between the patients with stage 2 and 3 hypertension (*P* > 0.05), and the same condition was also observed between the patients with stage 1 hypertension and without hypertension (*P* > 0.05).

Furthermore, in the patients over 73.6 years, the cutoff value of PNI for predicting PD after primary TJA is 46.99 (AUC: 0.679; 95 %CI: 0.597–0.778; sensitivity: 64.7 %; specificity: 72.7 %), and the cutoff value of PNI in patients under 73.6 years is 47.86 (AUC: 0.683; 95 %CI: 0.604–0.762; sensitivity: 60.6 %; specificity: 66.5 %). In addition, there is an obvious linear relationship between the PNI and PD (severity: *r* = -0.35, *P* < 0.05; duration: *r* = -0.43, *P* < 0.05), and there is no linear and non-linear relationship between age and both duration and severity of PD in this study (*P* > 0.05).

## Discussion

The significant prevalence of PD in this study indicates that almost one in fifteen patients undergoing primary TJA will develop PD, and similar and even higher prevalence was reported in many previous studies [4–6]. Recently published studies demonstrated an obvious correlation between PD and perioperative mortality [5, 18]. Therefore, clinicians should be aware of this so that they can pay more attention to preventing this condition when performing TJA surgery.

The results of this study demonstrated that malnutrition is one of the independent risk factors associated with PD following primary TJA, and a similar result has been reported in several previous studies [13, 14]. However, this view is still controversial, which may be ascribed to the different criteria for defining malnutrition [9]. Although a recently published meta-analysis demonstrated that serologic malnutrition is better than both anthropometric measurements and standardized nutrition score [9], there are still a large number of different serological indicators (e.g., albumin, transferrin, prealbumin and lymphocyte count) used to evaluate malnutrition [19–22]. Recently, Oe et al. reported that malnutrition identified by preoperative PNI was obviously associated with delirium after adult spinal deformity surgeries compared the malnutrition identified by other serologic indicators [12], and similar result was also identified in this study. A recent study further suggested that the onset of PD may be related to the postsurgical inflammatory or infectious changes [23], which was further supported obviously more patients in the PD group showing postoperative SSI in this study, and lymphocyte count is one of the important postoperative inflammatory indicators. Therefore, compared with using a single indicator, such as serum protein, the PNI, which combines both serum albumin and peripheral lymphocytes, may be a better index to quantify malnutrition, and it is important to assess the patient’s nutritional status using the preoperative PNI score to predict PD. Within our knowledge, it is the first time to investigate the role of malnutrition identified by PNI in predicting the PD after TJA.

In the present study, increased age was demonstrated to be associated with a higher risk of PD after TJA, and the impact of advanced age on PD has been widely reported, Brown et al. demonstrated that 40.5 % patients over 70 years developed delirium [24], and both Chen et al. and Peng et al. reported that approximately 20 % patients older than 65 years developed it after TJA [5, 6]. According to the previous studies, gradual accumulation of permanent damage to dendrites, neurons, microglia and receptors in elderly patients may be possible reason [25, 26], which make the elderly patients more susceptible to PD and cognitive impairment when biologically impairments.

Consistent with the previous studies [27, 28], the present study demonstrated that hypertension was strongly associated with both incidence and severity of PD following TJA, and this condition was especially in patients with moderate to severe hypertension. According to the previous studies [29, 30], hypertension is significantly associated with increased endothelial dysfunction and atherosclerosis, thus leading to an increased risk of cerebral embolization. As a result, the inhibited cerebral blood flow caused by cerebral atherosclerosis and postsurgical inflammatory changes results in postoperative PD [23]. Therefore, strict control of blood pressure and appropriate use of vascular protection and anticoagulant drugs are critical to reduce PD in the perioperative period of TJA.

The findings of this study should be interpreted with caution. It is not possible to predict PD by these independent risk factors alone because the mechanism leading to PD following TJA remains unclear. Thus, further study is required to identify the pathophysiological mechanism of PD and to establish the most effective strategy for PD prediction and prevention. Another clinical limitation of this study is that this is a retrospective study from a single center, which may limit our ability to deduce causal relationships and cause selection bias in patient enrollment. Therefore, we tried to minimize this bias by enrolling consecutive patients. Furthermore, multivariable logistic model is the main statistical method used in this study, and this data-driven model has relatively higher signal-to-noise ratio and is difficult to reflect the causal relationship. Therefore, more significant results might be achieved in a well-designed prospective study with a large sample size using a combination of both data-driven and knowledge-driven models.

## Conclusions

The present study demonstrated that age (> 73.5 years), hypertension, and malnutrition identified by PNI values (< 47.05) are the independent risk factors associated with the development of PD after primary TJA. Low preoperative PNI values should be cautioned about the risks of PD, and it is suggested that perioperative nutritional intervention to reduce the incidence of PD following primary TJA.

## Supplementary Information

**Additional file 1.**

## Data Availability

All measurements were collected from the patients accepting TJA recruited at the Huashan Hospital between January 2013 and October 2019, and the raw data of the current study are available from the corresponding author (Dr.xiajun@139.com) on reasonable request.

## Abbreviations

PNI: Prognostic nutritional index
TJA: Total joint arthroplasty
TKA: Total knee arthroplasty
THA: Total hip arthroplasty
PD: Postoperative delirium
MMSE: Mini-mental state examination
ASA: American Society of Anesthesiologists
BMI: Body mass index
SSI: Surgical site infection
OR: Odds ratio
CI: Confidence interval
CV: Cutoff
AUC: Area under the curve
ROC: Receiver operating characteristic
