# Supplementary material for: Preoperative prognostic nutritional index is useful factor for predicting postoperative delirium after primary total joint arthroplasty
Source: BMC Musculoskelet Disord. 2021 Sep 12;22:778. doi: 10.1186/s12891-021-04626-6 (PMC8436555; doi:10.1186/s12891-021-04626-6)
Supplement: Supplementary file 1 — Additional file 1. [file 12891_2021_4626_MOESM1_ESM.docx]

Patient questionnaire

| Name |  | Sex |  | Age |  |
| --- | --- | --- | --- | --- | --- |
| Height |  | Weight |  |  |  |
| 1. Do you often drink alcohol? How much wine do you drink every day? | | | | | |
|  | | | | | |
|  | | | | | |
|  | | | | | |
|  | | | | | |
| 1. Do you smoke a lot? How many cigarettes do you smoke every day? | | | | | |
|  | | | | | |
|  | | | | | |
|  | | | | | |
|  | | | | | |
| 1. What other diseases do you have? Whether to take medicine for this, how to use it? | | | | | |
|  | | | | | |
|  | | | | | |
|  | | | | | |
|  | | | | | |
| 1. Have you experienced any other surgical treatment? Specific operation and time? What problems are the operation and after surgical treatment? | | | | | |
|  | | | | | |
|  | | | | | |
|  | | | | | |
|  | | | | | |
